# Further Tests of Belief-Importance Theory

**DOI:** 10.1371/journal.pone.0121978

**Published:** 2015-04-13

**Authors:** K. V. Petrides, Adrian Furnham

**Affiliations:** 1 London Psychometric Laboratory, University College London (UCL), 26 Bedford Way, London, WC1H 0AP, United Kingdom; 2 Research Department of Clinical, Educational, and Health Psychology, University College London (UCL), London, United Kingdom; University of Bologna, ITALY

## Abstract

Belief-importance (belimp) theory hypothesizes that personality traits confer a propensity to perceive convergences or divergences between the belief that we can attain certain goals and the importance that we place on these goals. Belief and importance are conceptualized as two coordinates, together defining the belimp plane. We tested fundamental aspects of the theory using four different planes based on the life domains of appearance, family, financial security, and friendship as well as a global plane combining these four domains. The criteria were from the areas of personality (Big Five and trait emotional intelligence) and learning styles. Two hundred and fifty eight participants were allocated into the four quadrants of the belimp plane (Hubris, Motivation, Depression, and Apathy) according to their scores on four reliable instruments. Most hypotheses were supported by the data. Results are discussed with reference to the stability of the belimp classifications under different life domains and the relationship of the quadrants with the personality traits that are hypothesized to underpin them.

## Introduction

**Fig 1:**
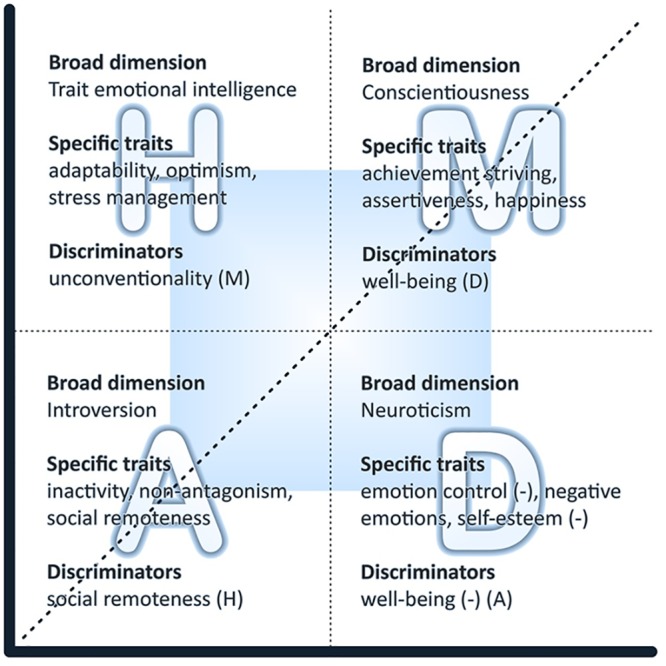
The Belief-Importance (Belimp) Plane. The figure presents the four belimp quadrants (Hubris, Motivation, Depression, and Apathy), along with the personality dimensions and specific personality facets that may underpin them. Because dimensions and facets will often cut across quadrants, we present, for each quadrant, a discriminating trait that helps distinguish it from adjacent quadrants (see [1] for empirical results). The axis of symmetry (diagonal line) and the inner and outer belimp plane regions (shaded and unshaded, respectively) are also depicted in the figure (see [2] for an explanation and discussion).

Belief-importance theory (abbreviated to ‘belimp’ in order to conserve space and facilitate the nomenclature) posits that certain personality traits confer on the individual a propensity to perceive convergences and divergences between their *belief* that they can attain goals and the *importance* that they place on these goals [1–4]. Belief and importance are conceptualized as two coordinates, together defining the *belimp plane* (see Fig 1). Although they are depicted as orthogonal, in practice, the two coordinates will generally be correlated because people tend to invest in goals that they value more.

Aspects of, mainly, conscientiousness and introversion confer a tendency to move *towards* the belimp *axis of symmetry* (see Fig 1), while aspects of, mainly, neuroticism and trait emotional intelligence (trait EI) confer a tendency to move *away* from the axis. Divergence from the axis creates discrepancies (residuals) that can be either *positive* (belief > importance) or *negative* (belief < importance). It is hypothesized that personality traits determine both the individual’s location along the axis of symmetry (high versus low) as well as the direction of the discrepancies (positive versus negative).

The two belimp coordinates (viz., belief and importance) are individually and jointly exposed to the effects of personality traits. Despite pronounced differences in value hierarchies, the theory posits that certain traits (e.g., aspects of conscientiousness) predispose people towards taking life more seriously and, thus, towards placing fairly high importance on multiple life domains (attractiveness, family, security, work, etc.). Life domains can be construed as intelligible regions of life experience [5]. The precise meaning, nature, and role of life domains in the context of belimp theory are discussed in Petrides [2]. Contrary to the view that confidence is essentially task-dependent [6], we believe that certain personality traits (especially aspects of trait EI) predispose people towards being generally confident.

Four quadrants are conceptualized within the belimp plane and, for heuristic purposes, labeled in terms of affect and motivation (see Fig 1). Clockwise from top left, we have the quadrants of Hubris, Motivation, Depression, and Apathy, loosely corresponding to the personality dimensions of trait EI, conscientiousness, neuroticism, and introversion. The labels are heuristic and intend to highlight connections between belimp processes and broad dimensions of personality. These connections relate primarily to specific facets of the dimensions, and not necessarily to their global scores, which often represent an amalgamation of rather disparate constructs [7].

Two different types of belimp plane can be identified: the *conditional* belimp plane, of which there are many, and the *master* belimp plane, of which there is only one. The former are planes specified in relation to a particular life domain (e.g., family or finance) and, therefore, conditional upon it. The latter is a hypothetical plane arising from averaging conditional planes over multiple life domains. An individual’s position in the master belimp plane represents their typical belimp position. Conditional belimp planes can be either concordant or discordant in relation to the master belimp plane and, more implicatively, in relation to a criterion. The degree of concordance between a conditional plane and the master plane is an empirical question (largely depending on the individual’s value hierarchy).

Because personality dimensions encompass quite heterogeneous concepts (for example, Extraversion combines sociability, activity, and impulsivity), no single personality dimension can be conceived of as the preserve of any one belimp quadrant. We may not presume that scores on, say, introversion-related variables will be statistically significantly higher in the Apathy quadrant than in the other three quadrants in every conditional plane, since aspects of introversion may well be implicated in all four quadrants. Nevertheless, we would expect that, over a number of randomly drawn life domains, pooled introversion scores in the Apathy quadrant will be at least numerically higher than in the other quadrants.

Central in belimp theory is the hypothesis that a person’s position in a conditional plane will be a function of their personality, the life domain under consideration, and other, undetermined, factors of more minor influence. Averaging over multiple conditional planes will cause all effects gradually to cancel out, except those of the dominant personality traits that are expected to act as principal determinants of the individual’s typical position in the master plane (from which positions in conditional planes will deviate to various extents).

### Significance of belimp theory and links with related theories

The significance of belimp theory lies in its potential to provide an explanatory mechanism for the effects of personality traits. One of the sharpest criticisms levelled against personality trait theory is that its explanations are often aetiologically weak or tautological [6]; [8]; [9]. In other words, there are hardly any mechanisms (e.g., in the sociocognitive sense) describing how personality traits come to affect real-life behaviors. Belimp theory offers such a mechanism based on convergences and divergences between how confident we are that we can achieve goals and how important these goals are to us.

For example, we propose that one specific process through which the personality dimension of neuroticism generates negative affect and, potentially, depression is through its tendency to produce discrepancies between the importance an individual attaches to certain goals and her confidence that she can achieve those goals. To be sure, these discrepancies will vary in their extent and manner of manifestation across different areas of life (life domains), but the neurotic person will be characterized by a generalized tendency to perceive discrepancies, rather than convergences, in their life. There are other noteworthy advantages of belimp theory, such as the efficiencies and improvements it affords in relation to criterion prediction, but these are outside the scope of the present paper and discussed elsewhere (e.g., [4]).

Belimp theory may be seen as a generalization of a class of theories known as expectancy—value (EV) theories (e.g., [10]; [11]). While the belief and importance coordinates could be roughly aligned to the concepts of expectancy and value, respectively, they are certainly not interchangeable with them. A description of the similarities and differences between belimp theory and EV theories is presented in Petrides [2].

Belimp theory, especially its belief coordinate, also intersects with Bandura’s [6] self-efficacy theory. Self-efficacy differs from the belimp coordinate of belief in that it is task-specific, rather than general, and concerns confidence in performing particular actions, rather than confidence in achieving broad goals. As previously noted, we believe that certain personality traits (e.g., aspects of trait EI) predispose people towards being generally confident that they can achieve, irrespective of the goals they are pursuing (although the confidence level will obviously vary from goal to goal). This means that, in general, individuals who are high on certain aspects of trait EI will be more likely to agree with statements starting “I really believe I can …”, irrespective of how they are completed (e.g., “…attain financial security,” “…be attractive,” or “…do well at school”). Thus, while self-efficacy is fully contextualized, the belief dimension in belimp theory is only partially contextualized in conditional planes and fully decontextualized in the master plane.

### Results of previous tests of belimp theory

A number of belimp studies have been conducted so far, the results of which are briefly summarized below. Petrides [1] tested 12 hypotheses based on a conditional belimp plane of attractiveness with reference to the Big Five and trait EI, and found support for ten of them. Petrides ([3]; Study 1) tested seven hypotheses based on a conditional belimp plane of financial security with reference to trait EI, mood, and somatic complaints, and found support for six of them, while in Study 2 he tested a total of nine hypotheses based on two conditional belimp planes (appearance and popularity) with reference to trait EI and also found support for six of them.

Petrides and Frederickson [4] tested eight hypotheses based on a conditional belimp plane of academic achievement with reference to Eysenckian personality [12], trait EI, and academic performance in national examinations, and found support for six of them. A set of hierarchical regressions in the same paper showed that the belimp co-ordinates (especially the coordinate of belief) were stronger predictors of academic performance than the Giant Three [12] personality dimensions.

### The present study

The present study seeks to replicate and extend the findings summarized above. Its major contribution is that it utilizes the largest number of conditional belimp planes so far (four: appearance, financial security, family, and friends). This will allow a direct investigation of the extent to which seemingly unrelated conditional planes produce convergent belimp classifications, as posited by the theory.

In addition, it will be possible to test the same hypotheses through different conditional belimp planes. The results of these tests are crucial for belimp theory because they must remain relatively invariant across the different conditional planes. In other words, the hypotheses advanced to test belimp theory are based solely on the individual’s location on the master belimp plane and do not get adjusted as a function of the life domain.

The study focuses on the following dependent variables: Big Five, trait EI, and learning approaches. With respect to the Big Five, we used the NEO-FFI, from which we derived item-cluster scores in addition to dimensional scores, as proposed by Saucier [13]. The Big Five personality dimensions are thought by some to provide a comprehensive operationalization of individual differences in human personality. They comprise Neuroticism (e.g., anxiety, moodiness, and vulnerability), Extraversion (e.g., activity, assertiveness, and talkativeness), Openness-to-Experience (e.g., curiosity, imagination, and originality), Agreeableness (e.g., cooperation, friendliness, and trust), and Conscientiousness (e.g., dependability, orderliness, and reliability). A detailed description of the Big Five model of personality can be found in McCrae and Costa [14].

Trait EI is defined as a constellation of emotional perceptions located at the lower levels of personality hierarchies [15]. Essentially, the construct concerns people’s perceptions of their emotional abilities. The global trait EI score provides a snapshot of one’s general emotional functioning. Among a host of other variables, it correlates positively with narcissism [16], which renders it relevant to the Hubris quadrant in the belimp plane (see Fig 1).

Learning approaches collectively refer to *why* learners learn (learning motives) as well as to *how* they like to learn (learning strategies). These motives and strategies give rise to broader learning approaches that describe students’ general orientation to learning ([17]; [18]). Three such approaches have been identified and studied in the literature: the surface approach (seeking to satisfy minimum requirements), the deep approach (seeking to understand what is learnt), and the achieving approach (seeking to maximize self-esteem through learning).

In accordance with belimp theory as outlined above, the following hypotheses were advanced:

*H1a*: The Hubris quadrant will have the highest score on trait EI

*H1b*: The Hubris quadrant will have the highest score on positive affect

*H2a*: The Motivation quadrant will have the highest score on conscientiousness

*H2b*: The Motivation quadrant will have the highest score on goal-striving

*H2c*: The Motivation quadrant will have the highest score on orderliness

*H3a*: The Depression quadrant will have the highest score on neuroticism

*H3b*: The Depression quadrant will have the highest score on negative affect

*H3c*: The Depression quadrant will have the highest score on self-reproach

*H4a*: The Apathy quadrant will have the lowest score on extraversion

*H4b*: The Apathy quadrant will have the lowest score on activity

*H4c*: The Apathy quadrant will have the lowest score on sociability

*H4d*: The Apathy quadrant will have the lowest score on the deep learning approach

*H4e*: The Apathy quadrant will have the lowest score on the achievement learning approach

*H5*: There will be a statistically significant overlap between the classifications derived from the four different life domains (appearance, family, finance, and friends).

It should be noted that these hypotheses do not imply that the target quadrant (e.g., Hubris in H1a) will score statistically significantly higher than all three other quadrants. Rather, the expectation is that the target quadrant will more likely have a numerically higher (or lower) score than the other three quadrants, if a hypothesis is fully supported, and a numerically higher (or lower) score than at least two other quadrants, if a hypothesis is partially supported. That is not to say, of course, that numerous statistically significant differences may not emerge anyway, especially in research designs with larger sample sizes (for a brief discussion of the role of sample size in belimp theory, see ‘Data analysis’ in the Method section).

## Method

### Participants

Two hundred and fifty eight individuals (mainly undergraduate psychology students at British universities) participated in the study. Demographic data were available for 190 (134 female and 56 male) participants, whose mean age was 19.68 years (SD = 2.43 years; range 18–42 years). Due to experimenter error, demographic data were missing from 68 participants in our sample and it is only possible to provide a general description of their characteristics. Thirty nine of the missing cases were undergraduate psychology students at a British university and would have had very similar characteristics to those reported above. The remaining 29 were post-graduate students in a business school and would have been somewhat older and roughly equally split on gender.

### Measures

#### Belimp instrument

Four life domains were assessed via standard belimp instruments (see S1 Questionnaires). The first instrument comprised five questions concerning the belief that certain appearance-related goals can be attained (“I really believe I can be good-looking”) and five matching questions concerning the importance placed on those goals (“It is important to me to be good-looking”). The alphas for the two scales were, respectively, .95 and .91.

The second instrument comprised five questions concerning the belief that certain family-related goals can be attained (“I really believe I can have a good relationship with my family”) and five matching questions concerning the importance placed on those goals (“It is important to me to have a good relationship with my family”). The alphas for the two scales were, respectively, .84 and .87.

The third instrument comprised five questions concerning the belief that certain finance-related goals can be attained (“I really believe I can be financially secure”) and five matching questions concerning the importance placed on those goals (“It is important to me to be financially secure”). The alphas for the two scales were, respectively, .86 and .75.

The fourth and last instrument comprised five questions concerning the belief that certain friends-related goals can be attained (“I really believe I can have a good relationship with my friends”) and five matching questions concerning the importance placed on those goals (“It is important to me to have a good relationship with my friends”). The alphas for the two scales were, respectively, .87 and .85.

#### Neuroticism Extraversion Openness—Five Factor Inventory (NEO-FFI; [19])

The NEO-FFI is a shortened version of the NEO Personality Inventory- Revised. It comprises 60 items, 12 for each of the five dimensions of adult personality: Neuroticism, Extraversion, Openness to Experience, Agreeableness, and Conscientiousness. The internal consistencies of the five factors were .83 for Neuroticism, .71 for Extraversion, .70 for Openness, .77 for Agreeableness, and .71 for Conscientiousness. In addition to scores on the five higher-order factors, we also derived item-cluster scores following the procedures outlined in Saucier [13]. The following clusters were used (internal consistencies are given in parentheses, followed by the initial of the Big Five dimension to which the cluster belongs): positive affect (.48—E), goal-striving (.71—C), orderliness (.43—C), negative affect (.65—N), self-reproach (.83—N), activity (.60—E), and sociability (.57—E).

#### Study Process Questionnaire (SPQ; [17])

The 42-item version of the SPQ was used to measure approaches to learning (surface, deep, and achieving). Items were responded to on a 5-point Likert scale. On this sample, the internal consistencies were .74, .65, and .73, respectively, for the surface, deep, and achieving learning approaches.

#### Trait Emotional Intelligence Questionnaire—Short Form (TEIQue-SF; [20])

This is a 30-item questionnaire designed to measure global trait EI. Two items from each of the 15 facets of the full form of the TEIQue were selected for inclusion, based primarily on their correlations with the corresponding total facet scores. A detailed description of the psychometric properties of the TEIQue-SF can be found in Cooper and Petrides [21]. The current study focuses on the global trait EI score, whose internal consistency was .85 on this sample.

### Procedure

Participants were given a battery of paper-and-pencil questionnaires, which they completed in class or in their own time. After the information was collected, data were anonymized through a coding system. Instructions were presented directly on the questionnaires and participation was voluntary. Informed consent was incorporated into the questionnaire. The study received ethical clearance by the ethics committee at the UCL Division of Psychology and Language Sciences.

### Data analysis

There are several different strategies for testing belimp theory, which Petrides [2] describes in some detail. In this paper, we will rely on one-way ANOVAs followed by post-hoc tests because of their simplicity and comparatively lower sample size requirements. Four groups will be formed based on 2x2 classification tables combining high and low scores on the two coordinates of belief and importance for each of the four life domains included in the study (appearance, family, finance, and friends). In addition, a global classification will be produced by combining ratings on the four aforementioned life domains.

As regards statistical power, the effects of personality traits on classifying participants into conditional belimp planes are expected to be small (not unlike the effects observed in molecular genetic studies of personality; [22]). This is due to the fact that belimp classifications are influenced by a multitude of other factors, especially by the life domain on which a conditional plane is based. Belimp theory is asymptotic [2], therefore, samples sizes much larger than the one employed herein will be required to obtain statistically significant results for every hypothesis tested. Nevertheless, numerical differences are of interest when evaluating belimp theory, particularly because they can be aggregated in the context of a meta-analysis.

In order to evaluate H5, viz., that there will be statistically significant overlap between the four different belimp classifications based on the life domains of appearance, family, finance, and friends, we will perform a series of chi-squared tests. With four different life domains, six cross-classifications are possible and, therefore, six chi-squared tests will be performed. Cramer’s V statistics, providing an indication of the strength of association between the variables, will also be reported.

## Results

Twelve one-way ANOVAs were performed per classification in order to test the bulk of the study hypotheses. Statistical details for the ANOVAs, including cell sizes, descriptive statistics, and Tukey post-hoc tests, are presented in Tables 1 to 5. The four groups in Fig 1 were derived in four different ways (i.e., separately for each life domain) by combining mean-split (high versus low) scores on belief and importance (1: appearance, 2:family, 3:finance, and 4:friends). A fifth classification (5: global) was derived from combined ratings on all four life domains. We present in the text a qualitative summary of the key findings under different subheadings corresponding to the five classifications.

**Table 1:** Descriptive Statistics and One-Way ANOVA Results for the Appearance Classification.

Variable	Hubris (h) n = 36		Motivation (m) n = 97		Depression (d) n = 43		Apathy (a) n = 82			Tukey post-hoc tests
	Mean	SD	Mean	SD	Mean	SD	Mean	SD	F	
Global trait EI	156.54	14.31	154.99	18.13	134.46	14.07	143.66	19.71	16.71*	m> a, d; h> d
Positive affect	14.14	2.09	14.34	2.08	13.52	2.84	14.52	2.80	1.62	**-**
Conscientiousness	42.06	8.27	43.95	6.41	43.24	4.89	44.10	5.45	.96	**-**
Goal-striving	11.08	2.35	11.94	1.91	11.84	1.84	11.38	2.03	2.26	**-**
Orderliness	16.67	3.76	16.89	3.12	16.60	2.39	17.00	2.86	2.05	**-**
Neuroticism	31.70	8.58	32.77	8.35	43.10	6.24	36.63	8.17	18.65*	a> h, m; d> a, h, m
Negative affect	15.21	3.85	15.28	3.56	17.67	4.06	15.99	3.49	4.68*	d>h, m
Self-reproach	16.29	5.24	17.54	5.00	24.10	4.11	19.93	5.29	21.49*	a> h, m; d> a, h, m
Extraversion	41.71	5.90	44.59	4.87	40.18	6.86	41.60	6.07	7.03*	m> d
Activity	13.91	2.82	14.64	2.17	13.26	2.45	13.43	2.64	4.81*	m> d
Sociability	13.80	2.39	14.47	2.24	13.60	2.91	13.08	2.62	4.62*	m> a
SDQ deep learning	47.00	6.57	48.23	8.89	45.39	6.84	46.19	7.82	1.54	**-**
SDQ achievement learning	45.09	8.68	47.98	6.21	47.03	6.10	46.35	7.90	1.55	**-**

*Note*. Degrees of freedom for all ANOVA numerators were 3 and for denominators ranged between 232–252, depending on missing data. Cell sizes indicate the number of participants allocated into the four belimp quadrants, but vary somewhat across analyses due to missing data on the dependent variables.

*p<.05.

**Table 2:** Descriptive Statistics and One-Way ANOVA Results for the Family Classification.

Variable	Hubris (h) n = 23		Motivation (m) n = 121		Depression (d) n = 38		Apathy (a) n = 77			Tukey post-hoc tests
	Mean	SD	Mean	SD	Mean	SD	Mean	SD	F	
Global trait EI	148.91	18.87	152.87	17.34	141.66	19.51	144.59	20.46	4.61*	m> a, d
Positive affect	14.81	2.38	14.03	2.12	14.11	3.03	14.47	2.70	.95	**-**
Conscientiousness	41.39	4.96	44.74	6.28	41.59	5.01	43.49	6.53	3.42*	m> d
Goal-striving	10.65	1.47	11.93	2.07	11.42	1.55	11.57	2.19	2.93*	m> h
Orderliness	15.58	2.76	17.30	3.08	15.92	2.39	16.93	3.10	3.33*	**-**
Neuroticism	33.91	6.93	34.11	8.45	38.89	9.13	36.47	9.32	3.38*	d> m
Negative affect	15.36	2.97	15.38	3.68	16.45	3.76	16.58	3.94	2.05	**-**
Self-reproach	18.83	5.04	18.29	5.17	21.21	6.22	19.81	5.74	3.14*	d> m
Extraversion	41.55	5.37	43.05	5.53	41.97	6.18	42.38	6.70	.58	**-**
Activity	13.23	1.74	14.13	2.48	13.89	2.39	13.86	2.80	.84	**-**
Sociability	13.57	3.13	14.03	2.20	13.39	2.80	13.73	2.80	.73	**-**
SDQ deep learning	47.59	7.91	47.01	8.48	46.97	6.01	46.88	8.20	.05	**-**
SDQ achievement learning	43.71	6.17	47.76	7.01	47.00	8.20	46.55	6.89	2.01	**-**

*Note*. Degrees of freedom for all ANOVA numerators were 3 and for denominators ranged between 232–253, depending on missing data. Cell sizes indicate the number of participants allocated into the four belimp quadrants, but vary somewhat across analyses due to missing data on the dependent variables.

*p<.05.

**Table 3:** Descriptive Statistics and One-Way ANOVA Results for the Finance Classification.

Variable	Hubris (h) n = 41		Motivation (m) n = 97		Depression (d) n = 58		Apathy (a) n = 64			Tukey post-hoc tests
	Mean	SD	Mean	SD	Mean	SD	Mean	SD	F	
Global trait EI	155.55	16.39	156.41	16.66	136.87	18.10	142.19	18.08	19.23*	h> a, d; m> a, d
Positive affect	14.95	2.09	13.80	2.38	14.04	2.80	14.63	2.39	2.85*	-
Conscientiousness	43.89	6.61	44.80	6.62	42.71	5.71	42.31	5.29	2.40	-
Goal-striving	11.88	1.87	12.01	2.05	11.29	1.97	11.22	2.00	2.81*	**-**
Orderliness	17.21	3.58	17.04	3.13	16.61	2.79	16.51	2.57	.68	**-**
Neuroticism	31.39	6.74	33.18	8.81	39.61	8.32	38.03	8.36	12.17*	a> h, m; d> h, m
Negative affect	15.10	3.72	15.40	3.49	17.94	15.69	15.69	3.62	4.93*	d> h, m, a
Self-reproach	16.20	4.07	17.88	5.48	21.44	5.20	21.08	5.46	12.79*	a> h, m; d> h, m
Extraversion	43.80	4.41	43.13	6.19	40.88	6.34	42.28	5.94	2.33	**-**
Activity	14.61	2.31	14.21	2.57	13.14	2.62	13.73	2.30	3.45*	h> d
Sociability	13.95	2.28	14.00	2.47	13.59	2.80	13.62	2.67	.47	**-**
SDQ deep learning	48.87	7.63	49.00	8.76	44.33	7.49	45.12	6.10	6.22*	h> d; m> d
SDQ achievement learning	45.68	9.54	48.69	6.95	45.96	6.33	45.95	5.80	2.99*	**-**

*Note*. Degrees of freedom for all ANOVA numerators were 3 and for denominators ranged between 233–254, depending on missing data. Cell sizes indicate the number of participants allocated into the four belimp quadrants, but vary somewhat across analyses due to missing data on the dependent variables.

*p<.05.

**Table 4:** Descriptive Statistics and One-Way ANOVA Results for the Friends Classification.

Variable	Hubris (h) n = 31		Motivation (m) n = 125		Depression (d) n = 29		Apathy (a) n = 75			Tukey post-hoc tests
	Mean	SD	Mean	SD	Mean	SD	Mean	SD	F	
Global trait EI	153.48	16.22	151.21	18.52	141.00	20.33	144.86	19.57	3.80*	m> d
Positive affect	14.74	2.50	14.23	2.51	14.11	2.63	14.10	2.33	.54	**-**
Conscientiousness	44.50	6.19	43.96	5.84	41.56	5.90	43.39	6.74	1.27	**-**
Goal-striving	12.03	1.80	11.76	1.81	11.41	2.16	11.35	2.33	1.17	**-**
Orderliness	17.03	3.05	16.92	2.85	16.19	3.12	16.88	3.21	.47	**-**
Neuroticism	35.07	6.83	34.46	8.80	39.93	8.17	35.51	9.46	3.08*	d> m
Negative affect	16.30	3.02	15.70	3.77	18.10	3.72	15.15	3.68	4.77*	d> a, m
Self-reproach	18.58	5.27	18.54	5.21	21.83	5.21	19.50	6.14	2.00	d> m
Extraversion	44.07	5.19	43.16	5.95	41.52	6.47	41.30	6.13	2.35	**-**
Activity	14.06	2.48	13.88	2.33	14.31	3.00	13.77	2.65	.35	**-**
Sociability	13.81	2.14	14.20	2.32	13.79	2.81	13.14	2.91	2.67*	m> a
SDQ deep learning	49.48	8.06	46.22	7.37	47.08	7.61	47.34	8.93	1.37	**-**
SDQ achievement learning	48.76	6.33	47.75	6.34	44.70	6.88	45.75	8.43	2.70*	**-**

*Note*. Degrees of freedom for all ANOVA numerators were 3 and for denominators ranged between 233–254, depending on missing data. Cell sizes indicate the number of participants allocated into the four belimp quadrants, but vary somewhat across analyses due to missing data on the dependent variables.

*p<.05.

**Table 5:** Descriptive Statistics and One-Way ANOVA Results for the Global Classification.

Variable	Hubris (h) n = 36		Motivation (m) n = 102		Depression (d) n = 48		Apathy (a) n = 72			Tukey post-hoc tests
	Mean	SD	Mean	SD	Mean	SD	Mean	SD	F	
Global trait EI	159.45	13.07	156.16	15.85	135.45	13.07	140.55	17.57	25.17*	h> a, d; m> a, d
Positive affect	14.40	2.44	14.27	2.12	13.21	2.85	14.79	2.53	2.05	a> d
Conscientiousness	43.90	7.76	44.33	6.39	42.36	6.30	43.28	5.96	1.12	**-**
Goal-striving	11.64	2.40	11.99	1.87	11.58	1.84	11.14	2.07	2.51	m>a
Orderliness	16.75	3.79	17.07	2.94	16.30	2.80	16.93	2.85	.71	**-**
Neuroticism	30.44	7.25	32.46	8.01	41.87	7.35	38.24	8.40	22.00*	a> h, m; d> h, m
Negative affect	14.91	3.66	15.32	3.54	17.73	3.72	15.97	3.75	5.73*	d> h, m
Self-reproach	15.34	4.49	17.46	4.68	22.87	5.19	21.24	5.34	23.78*	a> h, m; d> h, m
Extraversion	43.48	5.69	44.23	5.08	39.84	6.49	41.46	6.15	7.05*	h> d; m> a, d
Activity	14.53	2.56	14.33	2.28	13.62	2.54	13.25	2.66	3.53*	m> a
Sociability	14.00	2.24	14.38	2.10	13.19	2.84	13.24	2.93	3.94*	m > a, d
SDQ deep learning	48.91	7.25	48.13	8.78	45.67	7.73	45.20	6.90	2.94*	**-**
SDQ achievement learning	47.26	8.44	48.24	6.49	46.82	6.44	44.82	7.40	3.32*	m> a

*Note*. Degrees of freedom for all ANOVA numerators were 3 and for denominators ranged between 232–252, depending on missing data. Cell sizes indicate the number of participants allocated into the four belimp quadrants, but vary somewhat across analyses due to missing data on the dependent variables.

*p<.05.

### Appearance

Overall, six of the 13 hypotheses were supported fully, six partially, and one not at all (see Table 1). The Hubris quadrant had the highest score on trait EI, but only the third highest on positive affect, thus supporting *H1a* fully, but failing to support *H1b*. *H2a* and *H2c* were partially supported, with Motivation scoring second-highest on conscientiousness and orderliness. *H2b* received full support, with Motivation scoring highest on goal-striving. *H3a*—*H3c* were also fully supported, with the Depression quadrant scoring highest on neuroticism, negative affect, and self-reproach. Apathy had the second lowest score on extraversion, activity, and the deep and achievement learning strategies, thus partially supporting *H4a*, *H4b*, *H4d*, and *H4e*. It scored lowest on sociability, thus fully supporting *H4c*.

### Family

Overall, seven of the 13 hypotheses were supported fully, four partially, and two not at all (see Table 2). The Hubris quadrant had the second-highest score on global trait EI and the highest score on positive affect, thus providing partial support for *H1a* and full support for *H1b*. *H2a*—*H2c* were fully supported, with Motivation scoring highest on conscientiousness, goal-striving, and orderliness. *H3a* and *H3c* were also fully supported, with the Depression quadrant scoring highest on neuroticism and self-reproach. It scored second-highest on negative affect, thus partially supporting *H2b*. Apathy had the lowest score on the deep learning strategy, second-lowest score on activity and the achievement learning strategy, and third-lowest score on extraversion and sociability, thus fully supporting *H4d*, partially supporting *H4b* and *H4e*, and failing to support *H4a* and *H4c*.

### Finance

Overall, six of the 13 hypotheses were supported fully and seven partially (see Table 3). The Hubris quadrant had the second-highest score on global trait EI and the highest score on positive affect, thus providing partial support for *H1a* and full support for *H1b*. The Motivation quadrant had the highest score on conscientiousness and goal-striving, thus fully supporting *H2a* and *H2b*, and the second highest score on orderliness, thus partially supporting *H2c*. The Depression quadrant scored highest on neuroticism, negative affect, and self-reproach, thus fully supporting *H3a*—*H3c*. Apathy had the second-lowest score on all relevant dependent variables (viz., extraversion, activity, sociability, and the deep and achievement learning strategies), thus partially supporting *H4a—H4e*.

### Friends

Overall, eight of the 13 hypotheses were supported fully, four partially, and one not at all (see Table 4). *H1a* and *H1b* were fully supported, with the Hubris quadrant scoring highest on global trait EI and positive affect. The Motivation quadrant had the second highest score on conscientiousness, goal-striving, and orderliness, thus partially supporting *H2a*—*H2c*. *H3a*—*H3c* were fully supported, with the Depression quadrant scoring highest on neuroticism, negative affect, and self-reproach. Apathy had the lowest score on extraversion, activity, sociability, the second-lowest on the achievement learning strategy, and the third-lowest on the deep learning strategy. These results provided full support for *H4a*—*H4c*, partial support for *H4e*, and no support for *H4d*.

### Global

The global classification was derived from combining ratings on the aforementioned four life domains (see Table 5). Overall, ten of the 13 hypotheses were supported fully and three partially. The Hubris quadrant scored highest on trait EI and second-highest on positive affect, thus supporting *H1a* fully and *H1b* partially. *H2a*—*H2c* were fully supported with the Motivation quadrant scoring highest conscientiousness, goal-striving, and orderliness. *H3a*—*H3c* were also fully supported with the Depression quadrant scoring highest on neuroticism, negative affect, and self-reproach. Apathy had the lowest score on activity and the deep and achievement learning styles, and the second-lowest on extraversion and sociability. These results fully supported *H4b*, *H4d*, *and H4e* and partially supported *H4a* and *H4c*.

### Classification overlaps

The last hypothesis to be tested was *H5*, viz., that there will be statistically significant overlap between the four belimp classifications, which we assessed through six chi-squared tests.

Statistically significant associations were found between the appearance and family classifications (χ^2^_(9)_ = 18.05, p<.05; Cramer’s V = .153), the appearance and finance classifications (χ^2^_(9)_ = 72.44, p<.01; Cramer’s V = .530), the family and friends classifications (χ^2^_(9)_ = 38.74, p<.01; Cramer’s V = .223), the family and finance classifications (χ^2^_(9)_ = 22.82, p<.01; Cramer’s V = .171), and the finance and friends classification (χ^2^_(9)_ = 23.62, p<.01; Cramer’s V = .74). The only classifications that did not overlap significantly were appearance and friends (χ^2^_(9)_ = 8.00, p>.05; Cramer’s V = .102). Overall, these results provide strong support for *H5*.

## Discussion

This study employed the largest number of conditional belimp planes in the literature so far. Consequently, it allowed for the most rigorous investigation of central aspects of the theory, particularly those relating to the stability of belimp classifications.

### Stability of belimp classifications

Stability was directly assessed through a series of chi-squared tests designed to evaluate *H5*, viz., that the different belimp classifications will overlap. With four different life domains, six cross-classifications were possible, five of which showed statistically significant overlap.

For example, the classification of respondents into the four belimp quadrants (Hubris, Motivation, Depression, and Apathy) according to their ratings on the life domain of appearance overlapped significantly with their classification according to the life domain of finance. In other words, participants were fairly consistently classified into the same quadrants, irrespective of whether the allocation was based on appearance- or finance-related goals. And so for the classification pairs of family and friends, family and finance, and finance and friends. The only apparently independent classifications were appearance and friends.

These findings confirm and extend Petrides [3], wherein a strong overlap was recorded between two belimp classifications based on appearance and popularity. Our results go considerably beyond Petrides [3], however, not only in terms of the larger number of life domains involved, but also in terms of the spectrum of variation in the conceptual overlap of those domains. Thus, although it could be argued that the statistical overlap in Petrides [3] was partially a function of conceptually cognate life domains (appearance and popularity), this limitation was eliminated from our research design by simultaneously incorporating life domains that are not simply conceptually unrelated, but indeed potentially adversative (e.g., friends and finance).

Having established the counterintuitive, given the diverse nature of the life domains in our research design, overlap of the various cross-classifications, we may proceed to examine some of the underlying factors responsible for this overlap. Why is it that cross-classifications based on qualitatively different life domains should show any overlap at all? For such overlap to be documented, it is logically necessary that causal influences be in operation. Belimp theory [2] suggests that such influences are exerted by various underlying personality traits as described below.

### Personality traits and belimp quadrants

At the heart of belimp theory lies the premise that certain personality traits confer a predisposition to perceive convergences/discrepancies between an individual’s belief they can attain certain goals and the importance they place on these goals. These predispositions operate irrespective of the nature of the goals, although they may obviously be moderated by a host of other factors, such as interests, values, and personal circumstances. Fig 1 is an attempt to label those convergences/discrepancies and map them onto four broad personality traits.

How well do the results of this study support the mapping in Fig 1? Of a total of 52 different hypotheses based on four different life domains (13 hypotheses per domain), 27 were fully supported, 21 partially supported, and only four were not supported at all. When the ratings from the four life domains were combined into a global classification in order to attenuate the influences of extraneous factors, ten hypotheses were fully supported and three partially.

We believe that these results lend solid support to belimp theory, especially in light of the fact that most dependent variables had modest-to-low internal consistencies. Low alphas introduce error in the statistical analysis and attenuate the effects of independent variables. Therefore, we are confident that even more favorable results may be obtained with measures that are highly reliable.

Of the 25 hypotheses that were not fully supported, 15 (60%) involved the quadrant of Apathy. The Apathy quadrant is hypothesized to relate specifically to the trait of inactivity-activity, but, more generally, to the personality dimension of Introversion-Extraversion. From the present results, the trait of inactivity-activity does not seem to be a sufficiently strong marker to support hypotheses extrapolated to the entire dimension of Introversion-Extraversion. This may be especially true in operationalizations that under-emphasize the activity component of Extraversion, such as the Eysenckian (see, e.g., the testing of Hypothesis H4 in [4]).

A conceptual adjustment may also be required in relation to the Hubris quadrant, which is perhaps more closely related to narcissism than to trait emotional intelligence. As a key characteristic, the Hubris quadrant entails a conviction that one can attain even those goals that are unimportant to her, a fairly common belief among narcissists [23]. It is mainly the narcissistic aspect of trait EI (see [16]) that renders it a suitable candidate for underpinning the Hubris quadrant. Greater precision, and results that are even more promising, may be obtained by narrowing down some of the personality dimensions in belimp theory to primary personality traits (‘facets’). Such adjustments would be consistent with empirical findings showing that facets are often better predictors of behavioral outcomes than broad-bandwidth personality dimensions (e.g., [24]; [25]).

From the perspective of individual life-domains, appearance and finance yielded the fewest full confirmations of hypotheses (46%), while friends yielded the most (62%). Importantly, as far as the belimp principle of aggregation is concerned, the highest confirmation rate, overall, was achieved through the global classification (which pulled data from all four life domains), wherein 77% of hypotheses were fully supported. In line with belimp theory, this result clearly suggests that as data are pooled over multiple life domains, the relationship between the belimp quadrants and their underlying personality traits is strengthened (see paragraphs 5–7 in the introduction).

### Limitations

The student sample may have led to restriction of range effects due to the relative homogeneity of backgrounds and experiences. Restriction of range would have had a negative impact on the observed effects. Another shortcoming concerns the relatively low internal consistencies of many dependent variables that introduced error in the analyses, making it more difficult to find support for the study’s hypotheses. Last, the exclusive use of self-report questionnaires often undermines the differentiation between certain quadrants (especially between Motivation and Hubris). These quadrants may be more easily distinguishable with objective data (e.g., academic performance scores; see [4]).

## Conclusion

Belimp theory offers a general mechanism for linking personality traits to affect, motivation, and action. Setting aside its advantages in criterion prediction (e.g., [4]), a crucial benefit is that it describes a process through which the effects of personality traits are manifested in the world. This is theoretically valuable because it goes some way towards addressing the overwhelming explanatory deficits in personality trait theories [26].

Further possibilities present themselves in relation to personality interventions and behavior modification programs. Such interventions could explicitly target the belimp mechanism with a view to moderating the effects of the underlying traits without having to pursue the far more ambitious project of changing one’s standing on the traits themselves [27].

Overall, the results of the present study substantially reinforce previous findings, derived from related, but generally more limited, designs. Future research may continue to test and replicate core aspects of belimp theory, with particular emphasis on criterion prediction. In due course, this work may be able to provide a firm basis for the development of personality-driven interventions, along the lines suggested above.

## Supporting Information

S1 Dataset Belimp dataset for the study. (SAV)

S1 Questionnaires Belimp questionnaires for the study. (DOC)
