# Supplementary material for: Further Tests of Belief-Importance Theory
Source: PLoS One. 2015 Apr 13;10(4):e0121978. doi: 10.1371/journal.pone.0121978 (PMC4395152; doi:10.1371/journal.pone.0121978)
Supplement: S1 Questionnaires — (DOC) [file pone.0121978.s002.doc]

### BELIMP Questionnaires Used in the Study

### Part 1

INSTRUCTIONS FOR THE QUESTIONNAIRE

Below you will find five questions asking how important five financial goals are to you personally. In the box next to each question, please give a percentage score (anything from 0% to 100%) indicating importance. *0%* indicates that the goal is absolutely *unimportant* to you, whereas *100%* indicates that the goal is absolutely *important* to you. In other words, higher percentages indicate greater importance and lower percentages indicate less importance.

**It is important to me to…**

|  | Percentage |
| --- | --- |
| 1. …be financially independent. |  |
| 2. …have all the money I need. |  |
| 3. …achieve financial success. |  |
| 4. …be able to afford the things that I really desire. |  |
| 5. …be financially secure. |  |

The questions below ask *how confident* you are that you can reach the same five financial goals that you previously rated for their importance to you. Use the same percentage rating system as above (from 0% to 100%) to answer those questions. *0%* indicates *minimum* confidence that you can reach a particular goal, whereas *100%* indicates *maximum* confidence that you can reach a particular goal.

**I really believe I can…**

|  | Percentage |
| --- | --- |
| 1. …be financially independent. |  |
| 2. …have all the money I need. |  |
| 3. …achieve financial success. |  |
| 4. …afford the things that I really desire. |  |
| 5. …be financially secure. |  |

**Part 2**

**INSTRUCTIONS FOR THE QUESTIONNAIRE**

Below you will find five questions asking how important five friends-related goals are to you personally. Please use the same percentage rating system as in Part 1 above: *0%* indicates that the goal is absolutely *unimportant* to you, whereas *100%* indicates that the goal is absolutely *important* to you.

**It is important to me to…**

|  | Percentage |
| --- | --- |
| 1. …have a good relationship with my friends. |  |
| 2. …be available for my friends when they need me. |  |
| 3. …be fully involved with my friends. |  |
| 4. …communicate with my friends regularly. |  |
| 5. …be able to rely on my friends. |  |

The questions below ask *how confident* you are that you can reach the same five friends-related goals that you previously rated for their importance to you. Please use the same percentage rating system as above. *0%* indicates *minimum* confidence that you can reach a particular goal, whereas *100%* indicates *maximum* confidence that you can reach a particular goal.

**I really believe I can…**

|  | Percentage |
| --- | --- |
| 1. …have a good relationship with my friends. |  |
| 2. …be available for my friends when they need me. |  |
| 3. …be fully involved with my friends. |  |
| 4. …communicate with my friends regularly. |  |
| 5. …rely on my friends. |  |

### Part 3

INSTRUCTIONS FOR THE QUESTIONNAIRE

Below you will find five questions asking how important five appearance-related goals are to you personally. Please use the same percentage rating system as in Part 2 above: *0%* indicates that the goal is absolutely *unimportant* to you, whereas *100%* indicates that the goal is absolutely *important* to you.

**It is important to me…**

|  | Percentage |
| --- | --- |
| 1. …to look beautiful/handsome. |  |
| 2. …to get noticed for my good looks. |  |
| 3. …to have an attractive figure. |  |
| 4. …to be good-looking. |  |
| 5. …to be seen as attractive. |  |

The questions below ask *how confident* you are that you can reach the same five appearance-related goals that you previously rated for their importance to you. Use the same percentage rating system as above (from 0% to 100%) to answer those questions. *0%* indicates *minimum* confidence that you can reach a particular goal, whereas *100%* indicates *maximum* confidence that you can reach a particular goal.

**I really believe I can…**

|  | Percentage |
| --- | --- |
| 1. …look beautiful/handsome. |  |
| 2. …get noticed for my good looks. |  |
| 3. …have an attractive figure. |  |
| 4. …be good-looking. |  |
| 5. …be seen as attractive. |  |

### Part 4

INSTRUCTIONS FOR THE QUESTIONNAIRE

Below you will find five questions asking how important five family-related goals are to you personally. Please use the same percentage rating system as in Part 3 above: *0%* indicates that the goal is absolutely *unimportant* to you, whereas *100%* indicates that the goal is absolutely *important* to you.

**It is important to me…**

|  | Percentage |
| --- | --- |
| 1. …have a good relationship with my family. |  |
| 2. …be available for my family when they need me. |  |
| 3. …be fully involved with my family. |  |
| 4. …communicate with my family regularly. |  |
| 5. …be able to rely on my family. |  |

The questions below ask *how confident* you are that you can reach the same five family-related goals that you previously rated for their importance to you. Use the same percentage rating system as above (from 0% to 100%) to answer those questions. *0%* indicates *minimum* confidence that you can reach a particular goal, whereas *100%* indicates *maximum* confidence that you can reach a particular goal.

**I really believe I can…**

|  | Percentage |
| --- | --- |
| 1. …have a good relationship with my family. |  |
| 2. …be available for my family when they need me. |  |
| 3. …be fully involved with my family. |  |
| 4. …communicate with my family regularly. |  |
| 5. …be able to rely on my family. |  |
